# How Reliable Are Current Data for Assessing the Actual Prevalence of Chronic Obstructive Pulmonary Disease?

**DOI:** 10.1371/journal.pone.0149302

**Published:** 2016-02-22

**Authors:** Anna Maria Romanelli, Mauro Raciti, Maria Angela Protti, Renato Prediletto, Edo Fornai, Annunziata Faustini

**Affiliations:** 1 CNR, Institute of Clinical Physiology, Pisa, Italy; 2 Fondazione Gabriele Monasterio CNR-Regione Toscana, Pisa, Italy; 3 Department of Epidemiology, Regional Health Service, Lazio Region, Rome, Italy; University of Rochester Medical Center, UNITED STATES

## Abstract

**Background:**

Estimating COPD occurrence is perceived by the scientific community as a matter of increasing interest because of the worldwide diffusion of the disease. We aimed to estimate COPD prevalence by using administrative databases from a city in central Italy for 2002–2006, improving both the sensitivity and the reliability of the estimate.

**Methods:**

Multiple sources were used, integrating the hospital discharge register (HDR), clinical charts, spirometry and the cause-specific mortality register (CMR) in a longitudinal algorithm, to reduce underestimation of COPD prevalence. Prevalence was also estimated on the basis of COPD cases confirmed through spirometry, to correct misclassification. Estimating such prevalence relied on using coefficients of validation, derived as the positive predictive value (PPV) for being an actual COPD case from clinical and spirometric data at the Institute of Clinical Physiology of the National Research Council.

**Results:**

We found that sensitivity of COPD prevalence increased by 37%. The highest estimate (4.43 per 100 residents) was observed in the 5-year period, using a 3-year longitudinal approach and combined data from three sources. We found that 17% of COPD cases were misclassified. The above estimate of COPD prevalence decreased (3.66 per 100 residents) when coefficients of validation were applied. The PPV was 80% for the HDR, 82% for clinical diagnoses and 91% for the CMR.

**Conclusions:**

Adjusting the COPD prevalence for both underestimation and misclassification of the cases makes administrative data more reliable for epidemiological purposes.

## Background

The most recent estimate of chronic obstructive pulmonary disease (COPD) prevalence shows a global burden of the disease of 10.1% [1]. Estimating COPD occurrence is perceived by the scientific community as a matter of increasing interest because of the worldwide diffusion of the disease, the predicted increase in mortality and the deterioration of quality of life for COPD patients [2].

Many current registers of vital statistics, administrative databases such as mortality and hospital discharge registers, health insurance refunds and pharmaceutical data, have long been routinely used to estimate both impact and risk assessment of diseases in populations [3, 4]. Underestimation and misclassification of actual cases are the most important limitations of these databases, which may affect the estimation of disease occurrence as well as the fractions attributable to different factors [5]. Underestimation is partly due to the different probabilities with which patients have recourse to various health services, while misclassification is mostly due to misdiagnosis or registration errors. Misclassification is a possible consequence of specific faults in COPD diagnosis because of the deceptive onset of the disease, insufficient recourse to spirometric testing and the need for a differential diagnosis between it and other respiratory diseases.

Given the limits of current registers and the specific problems in estimating the COPD burden, validation of diagnoses is a prerequisite for using administrative databases for epidemiological purposes. Not many studies are available in the literature [6]; the first ones were oriented towards internal validation of single registers [7] and only a few of them attempted external validation, which was based on family doctors’ registers or questionnaires for patients [8, 9]. Validation estimates based on clinical and spirometric data were initially measured in COPD cohorts [10] or COPD population registers [11]. Finally, validation of COPD diagnoses was based on databases such as longitudinal medical records for primary care in the UK [12] or multiple administrative databases [13].

In this paper, we estimated the COPD prevalence, using an enhanced approach based on multiple registers and longitudinal estimates [14, 15] so as to reduce underestimation, and derived reliable coefficients of validation from clinical and spirometric data, which allowed us to correct misclassification of COPD cases.

## Methods

### Study population

A COPD case was defined as a 40-plus-year-old subject who had been discharged from hospital with a principal or secondary diagnosis of COPD, or who had received a diagnosis of COPD in clinical (hospital or outpatient) charts, or had shown a ratio of one-second forced expiratory volume (FEV1) to forced vital capacity (FVC) < 0.70 at spirometry [16], or a subject who died with COPD registered as an underlying cause of death.

COPD cases were obtained from Pisa, a city (88,627 inhabitants) in central Italy. The city’s hospital discharge register (HDR) and the cause-specific mortality register (CMR) were used as sources of data for the 2000–2006 period. Clinical and spirometric data were obtained from clinical (hospital or outpatient) charts for 2000–2006 at the Institute of Clinical Physiology (ICP) of the National Research Council (NRC). The Institute, located in Pisa, is a center for research into cardio-pulmonary disease.

Subjects did not participate in the study in person, since administrative and medical databases were used in accordance with the privacy laws in effect in Italy; clinical charts were consulted by researchers from the NRC upon approval by the Local Health Authority Ethical Committee denominated Comitato Etico Area Vasta Nord-Ovest Toscana. Patient records were anonymized and de-identified prior to analysis.

### COPD prevalence and underestimation

The prevalence of COPD was estimated by using all available records and a longitudinal approach [14,15] to reduce underestimation of COPD cases and to increase sensitivity of COPD prevalence. Prevalent COPD cases per one year were calculated including in sequence, all the COPD cases reported in the HDR in the course of the year of interest or during two (or four) previous years, if they were resident in Pisa and still alive on 1 January of the year of interest, the COPD cases who were diagnosed in hospital, those who received a COPD diagnosis in outpatient clinics or, finally, at spirometry at the NRC Institute, in the course of the year of interest or during two (or four) previous years (if they were resident in Pisa, were still alive on 1 January of the year of interest, and, in addition, had never been registered in the HDR); lastly, those who died from COPD in that year, were resident in Pisa and had never been registered in the HDR or clinical records at the NRC Institute during that year or the two (or four) previous years, were added. The algorithm for identifying the prevalent COPD cases is reported in detail in Table 1 for the period 2004–2006 (length of longitudinal period of both 3-year and 5-year) and the 2002–2006 period (longitudinal period of 3-year only). The codes of the International Classification of Diseases, 9^th^ revision (ICD-9) that we used to identify COPD cases in the HDR and the CMR are reported in S1 Table.

**Table 1:** Algorithm for enrollment of COPD cases and contributing to COPD prevalence, according to prevalence periods and length of longitudinal periods.

	prevalence periods		
	2002–2006	2004–2006	2004–2006
	length of longitudinal periods		
	3yrs	3yrs	3yrs
Definition of cases	2000–2006	2002–2006	2000–2006
Subjects with one of the HDR ICD9 codes (490, 491, 492, 494, 496) as principal or secondary diagnosis and still alive at the beginning of the prevalence period	2182	1654	1897
**+**			
Subjects with COPD diagnosis in hospital chart, still alive at the beginning of the period, with no HDR report in the longitudinal period	17	12	13
**+**			
Subjects with COPD diagnosis in outpatient clinic chart, still alive and with no HDR report or hospital chart in the longitudinal period	33	35	33
**+**			
Subjects with spirometry and FEV1/FVC < = 0.70, with no HDR report or clinical charts in the longitudinal period	250	246	247
**+**			
Subjects deceased with COPD as underlined cause in the prevalence period, with no HDR report or clinical charts or spirometry in the longitudinal period	62	38	33
=			
**COPD prevalent cases enrolled in each period**	**2544**	**1985**	**2223**

Underestimation of COPD cases or increase in sensitivity is estimated as the percentage of the additional cases seen by multiple sources, by a 3-year and 5-year longitudinal approach in sequence, compared with those seen in HDR only, with a cross-sectional approach.

Crude and age-standardized rates of prevalence were estimated, for both 2002–2006 and 2004–2006, as the percentage of COPD cases in the resident population as of 30 June for each year–with 95% confidence intervals (95% CI)–according to both cross-sectional and 3-year longitudinal approaches; data for 5-year longitudinal estimates were available only for 2004–2006. The 2006 Italian population divided into 5-year age groups was used to standardize rates by age. Subjects’ ages as given in the HDR or clinical records in previous periods were updated to the same day and month of the year of interest.

### COPD validation and misclassification

Confirmed cases were defined as those who showed a ratio of one-second forced expiratory volume (FEV1) to forced vital capacity (FVC) < 0.70 at the most recent spirometry [16], which they underwent at the NRC Institute, in the three months preceding or following the most recent recourse to a health service. All prevalent cases from HDR and clinical (hospital or outpatient) charts were assessed, whereas prevalent cases identified from spirometry registers all had, by definition, a FEV1/FVC ratio <0.7 and no prevalent case from the CMR could have had spirometry, since they were enrolled as prevalent cases only if they had not been seen by any other health service.

COPD cases confirmed at spirometry were analyzed by age group (40–49, 50–59, 60–69, 70–79 and 80+), gender and the COPD ICD-9 code recorded in the HDR, such as each single COPD ICD-9 code, the more specific codes (491, 492, 496) and the less specific ones (490, 494), all COPD codes at the principal diagnosis, or at the secondary diagnosis when the principal diagnosis was respiratory failure (ICD-9 codes 518.8, 518.5, 786.0), pneumonia (ICD-9 codes 480–487) or congestive heart failure (ICD-9 code 428.0), or at secondary diagnosis with principal diagnoses unrelated to COPD. An additional sensitivity analysis was carried out, using the Lower Limit of Normal (LLN) as a dynamic threshold of the FEV1/FVC ratio, to confirm the COPD cases.

Finally, prevalence was re-assessed after excluding misclassified COPD cases. Estimating such a prevalence in the population required the following steps: first of all, validation coefficients for each source of data were obtained by using all the COPD cases seen at the NRC Institute and registered in each database independently, whether they were present in other registers or not. All the spirometry tests were done at the same Institute, where the European Respiratory Society standards [17] for 40–69-year-olds and the American standards [18] for 70+-year-olds were used as reference values for pulmonary volumes. Then, the positive predictive value (PPV) for a confirmed COPD case was calculated as the ratio between positive and negative spirometry tests among all the cases registered as COPD in each source: HDR, hospital and outpatient charts, and the CMR. Thereafter, the respective coefficients were applied to the prevalent COPD cases from the HDR, the clinical charts and the CMR at city levels and finally prevalence was re-assessed on the basis of confirmed COPD cases.

## Results

In the 2002–2006 period, using a 3-year longitudinal approach, we found 2,544 prevalent COPD cases among 40-plus-year-old residents (Tables 1 and 2). In comparison with cross-sectional estimates based on the HDR, 20.3% additional cases emerged as a result of using multiple contemporary registers, and a further 14.3% from using a longitudinal approach Table 2.

**Table 2:** COPD cases and prevalence, by periods and method of estimation.

Method of estimating	2002–2006				2004–2006			
prevalence	N	%^a^	95% CI		N	%^a^	95% CI	
**cross-sectional, from HDR**	1850	3.16	3.15	3.18	1243	2.11	2.1	2.12
**cross-sectional, from all sources**	2225	3.83	3.82	3.85	1495	2.56	2.55	2.58
**3-yr longitudinal**	2544	4.43	4.42	4.45	1985	3.45	3.44	3.46
**5-yr longitudinal**	---	---			2223	3.87	3.86	3.89
								

COPD chronic obstructive pulmonary disease; 95% CI 95% confidence intervals.

^a^prevalence per 100 40+ year-old residents, standardized by age.

In the 2004–2006 period, prevalent COPD cases numbered 1985 and 2223, using 3-year and 5-year longitudinal approaches respectively. A 20.3% increase in cases was found thanks to using multiple registers; a further 32.8% was obtained when prevalent COPD cases from the previous two years were included, and an additional 12% when that period was extended to include four years Table 2.

Hospital registers contributed the most COPD cases, and spirometry registers came second Table 1. The number of cases from each source are shown in Table 3, either as all registered cases independently of their presence in other registers (absolute contribution) or as uniquely registered cases (exclusive contribution).

**Table 3:** Absolute / exclusive contribution to longitudinal prevalence of COPD by source, prevalence period and length of longitudinal period.

Prevalence periods	2002–2006		2004–2006		2004–2006	
Long-period length	(3-year)		(3-year)		(5-year)	
Sources of COPD	Absolute	Exclusive	Absolute	Exclusive	Absolute	Exclusive
cases	N	N	N	N	N	N
HDR	2182	1898	1654	1451	1897	1634
Ward charts^a^	40	17	25	12	40	13
Outpatient clinic charts^a^		33	58	35	60	33
Spirometric tests^a^	430	250	415	246	430	246
CMR	184	62	111	38	111	33

COPD chronic obstructive pulmonary disease; HDR hospital discharge register; CMR cause mortality register.

^a^from NRC hospital only.

**Fig 1:**
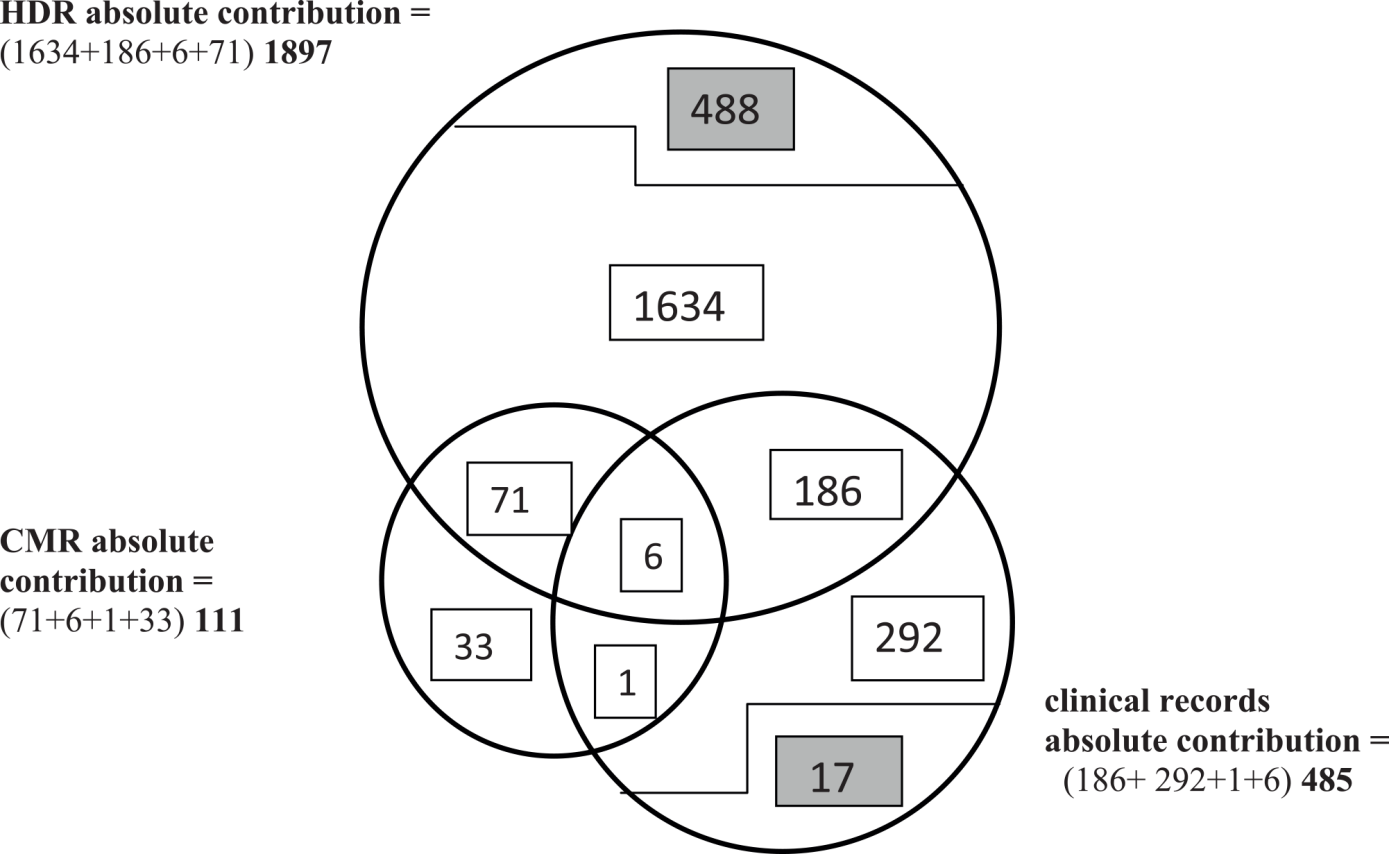
Non-proportional Venn diagram describing the absolute and the exclusive contributions in cases to COPD prevalence in 5-year longitudinal 2004–2006 period, by sources. **1634** subjects hospitalized (listed in HDR) in the longitudinal period, still alive at the beginning of the prevalence period, without clinical records at the NRC Institute: enrolled as **HDR exclusive contribution. 488 and 17** hospitalized subjects and outpatients respectively during 4 years preceding 2004, and deceased by the beginning of prevalence period: not included in the absolute contribution of CMR, HDR or clinical records since they had not been enrolled as prevalent cases. **186** subjects hospitalized (listed in HDR) in the longitudinal period, still alive at the beginning of the prevalence period, with clinical records also at the NRC Institute: enrolled as prevalent cases from HDR. **71** subjects deceased in the prevalence period, during or after hospitalization (HDR): enrolled as prevalent cases from HDR. **6** subjects deceased in the prevalence period, during or after hospitalizations (HDR), with clinical records as well: enrolled as prevalent cases from HDR. **292** subjects with COPD diagnosis in clinical charts or spirometry in the longitudinal period, not listed in HDR, still alive at the beginning of the prevalence period: enrolled as **clinical exclusive contribution. 1** subject deceased in the prevalence period, without hospitalization (HDR) but with spirometry: enrolled as prevalent case from clinical (spirometry) records. **33** subjects deceased in the prevalence period with COPD as underlying cause, without hospitalization (not listed in HDR) or clinical records during the longitudinal period: enrolled as **CMR exclusive contribution. Note: Clinical absolute contribution as reported here excludes those cases registered in common with other clinical sources.**

Comparing Table 1 with Table 3, one can see that the prevalent COPD cases, which are reported from the HDR in Table 1, correspond to the absolute HDR contribution in Table 3, since they include the COPD cases registered in the HDR as a unique source plus those registered in common with the following sources (clinical records and CMR). In contrast, the prevalent COPD cases which are reported from the CMR in Table 1 correspond to the exclusive CMR contribution in Table 3, since the CMR was the last step in the algorithm and the COPD cases this source has in common with the other sources have already been included. Finally, the numbers of prevalent cases reported from clinical records in Table 1 range between the absolute and exclusive contributions of each specific clinical source reported in Table 3, and approach the exclusive contribution of Table 3 as the enrollment moves forward in Table 1, and Fig 1 shows a Venn diagram of contributions to the 5-year-long prevalence in the period 2004–2006.

COPD prevalence amounted to 3.83% (95% CI 3.82%-3.85%) in 2002–2006, and 2.56% (95% CI 2.55%-2.58%) in 2004–2006, when it was calculated on the basis of a cross-sectional approach, but using all sources of data. Prevalence increased to 4.43% (95% CI 4.42%-4.45%) and 3.45% (95% CI 3.44%-3.46%) respectively when we used the 3-year longitudinal approach, showing a 37% underestimation of COPD prevalent cases in the cross-sectional estimates based on HDR only Table 2). The sensitivity analysis (based on the LLN threshold) showed a somewhat lower COPD prevalence; the 3-year longitudinal estimates equaled 4.22% (95% CI 4.20%-4.24%) in 2002–2006, and 3.25% (95% CI 3.23%-3.26%) in 2004–2006.

Longitudinal estimates were at least three times higher in men (7.51%; 95% CI 7.48%-7.55%) than in women (2.45%; 95% CI 2.43%-2.46%) in the longer period, but gender made less of a difference in the later period, 2004–2006, with estimates of 5.78% (95% CI 5.75%-5.81%) in men and 1.95% (95% CI 1.94%-1.97%) in women (data not shown). Prevalence increased with age in both periods, showing estimates of 0.60% in the youngest subjects and 14.31% in the oldest in 2002–2006, and of 0.52% and 10.81% respectively in 2004–2006 (data not shown).

**Fig 2:**
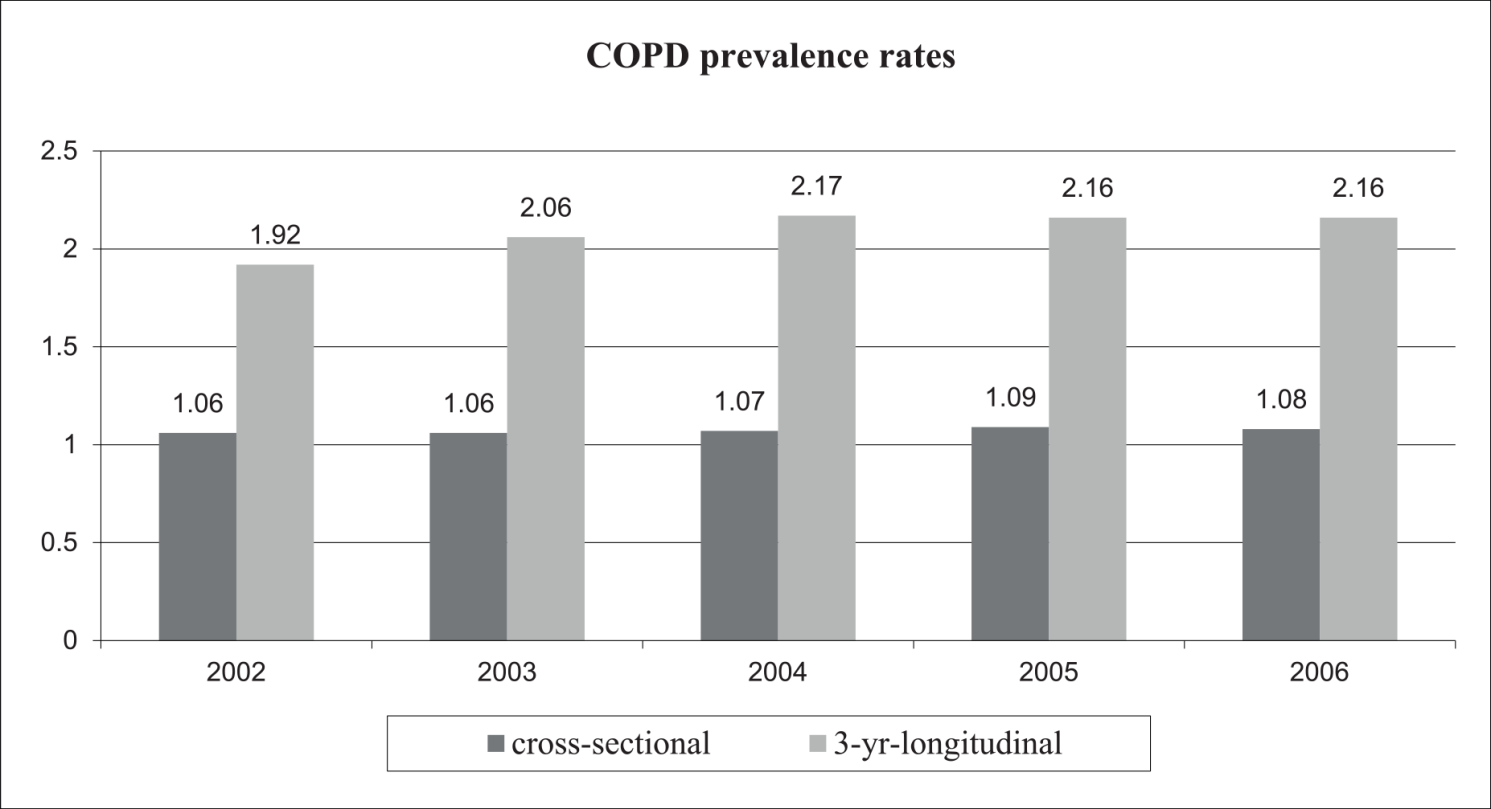
COPD prevalence from 2002 to 2006: Impact of longitudinal approach.

No important trend in COPD prevalence was observed between 2002 and 2006 with either cross-sectional or longitudinal rates, though the values tended to increase slightly. Longitudinal estimates were higher than cross-sectional ones for each year Fig 2).

Of the prevalent COPD cases, 19% and 23% had spirometry in 2002–2006 and 2004–2006 respectively Table 4. Recourse to the test did not differ substantially between women and men, whereas more 40–79-year-olds than 80+-year-olds were tested. These relationships persisted in the two prevalence periods. Of the COPD patients who had spirometry, 88% were confirmed in each period according to the FEV1/FVC ratio Table 4.These confirmed COPD cases showed similar percentages in men and women, but were more frequent among the youngest patients than among the 70+-year-olds Table 4. When the same prevalent COPD cases were confirmed according to the LLN threshold, the confirmed cases decreased by as much as 79% in the longer period (2002–2006) and 78% in 2004–2006, with no important differences between men and women. In both periods, they diminished in all age groups, showing the greatest decrease among the most elderly.

**Table 4:** Prevalent COPD cases with spirometry tests in two prevalence periods (3-year long longitudinal period), by sex, age group and COPD definitions, in 40+ year-old residents.

	**2002–2006**					
	**COPD cases**	**Spirometry- tested**		**FEV1/FVC < 0.7**		
	**N**	**n**	**%**	**n**	**%** ^a^	**mean**
**All prevalent cases**	**2544**	**474**	**18.6**	**415**	**87.6**	**0.59**
men	1681	325	19.3	285	87.7	0.58
women	863	149	17.3	130	87.2	0.61
40–49	73	22	30.1	20	90.9	0.59
50–59	181	47	26.0	40	85.1	0.60
60–69	495	125	25.3	114	91.2	0.59
70–79	936	200	21.4	175	87.5	0.59
80+	859	80	9.3	66	82.5	0.57
	**2004–2006**					
	**COPD cases**	**Spirometry- tested**		**FEV1/FVC < 0.7**		
	**N**	**n**	**%**	**n**	**%** ^a^	**mean**
**All prevalent cases**	**1985**	**460**	**23.2**	**403**	**87.6**	**0.59**
men	1299	315	24.2	275	87.3	0.59
women	686	146	21.3	128	87.7	0.61
40–49	65	22	33.8	19	86.4	0.60
50–59	154	44	28.6	38	86.4	0.60
60–69	369	124	33.6	113	91.1	0.60
70–79	725	189	26.1	166	87.8	0.58
80+	672	81	12.1	67	82.7	0.59
**ICD-9 COPD codes in HDR**	1638	158	*9*.*6*	112	*70*.*9*	
490	3	0				
491	1440	135	*9*.*4*	99	*73*.*3*	
492	80	9	*11*.*3*	5	*55*.*6*	
494	35	3	*8*.*6*	1	*33*.*3*	
496	80	11	*13*.*8*	7	*63*.*6*	
491, 492, 496^b^	1600	155	*9*.*7*	111	*71*.*6*	
490, 494^c^	38	3	*7*.*9*	1	*33*.*3*	
**principal diagnosis**	**274**	**26**	***9*.*5***	**17**	***65*.*4***	
**secondary diagnosis**	1364	132	*9*.*7*	95	*72*.*0*	
- with CHF, pneumonia or RF	**183**	**20**	***10*.*9***	**17**	***85*.*0***	
in principal diagnosis						
- with other diseases	**1181**	**112**	***9*.*5***	**78**	***69*.*6***	
in principal diagnosis						

COPD chronic obstructive pulmonary disease; FEV1/FVC one-second forced expiratory volume (FEV1) to forced vital capacity (FVC).

^a^ percentage of subjects tested by spirometry.

^b^ the more specific codes.

^c^ the less specific codes.

The confirmed cases in the hospital discharge register amounted to 71% in the 2004–2006 period (but only 10% of these 1638 hospitalized cases had spirometry at the NRC Institute). The diagnosis of chronic bronchitis (ICD-9 code 491) showed the highest percentage (73.3%) of confirmation among the single ICD-9 codes we used to identify COPD Table 4. The most confirmed diagnoses (85.0%, n.183 cases), however, were those combining a secondary diagnosis of COPD (any ICD-9 COPD code) with a principal diagnosis of respiratory failure (92.9%), pneumonia (50.0%) or heart failure (75%). Secondary COPD diagnoses with other principal diagnoses followed, with 69.6% (n.1,181 cases). Only two patients in this group had a principal diagnosis of asthma, but they did not have spirometry. Finally, confirmed principal diagnoses of COPD amounted to 65.4% (n. 274 cases).

Of the patients hospitalized or seen in outpatient clinics at the NRC Institute, 145 (76%) had spirometry in the 2002–2006 period Table 5. The positive predictive value for COPD diagnoses in the HDR was 80.2%; it was a bit higher for clinical diagnoses in hospital charts (82.4%) and outpatient charts (81.8%). The highest positive predictive value (90.9%) was observed for COPD as an underlying cause of death in the CMR Table 5.

**Table 5:** COPD cases confirmed on the basis of spirometry, by data source, in 40+-year-old residents, at NRC-institute, 2002–2006.

Sources of COPD cases	FEV1/FVC			
from NRC—institute	= < 0.7	> 0.7	Tot	PPV (%)
**HDR**	77	19	96	80.2
**ward charts**	14	3	17	82.4
**outpatient clinic charts**	45	10	55	81.8
**CMR**	10	1	11	90.9

COPD chronic obstructive pulmonary disease; FEV1/FVC one-second forced expiratory volume (FEV_1_) to forced vital capacity (FVC); HDR hospital discharge registry; CMR cause mortality registry PPV positive predictive value.

When these estimates were applied to the prevalent COPD cases as validation coefficients, up to 17% of COPD cases were unconfirmed; the contribution of cases diminished by 20.5% for the HDR, while that of deceased cases diminished only by 5.13%. The prevalence of validated COPD cases diminished in both periods, arriving at estimates of 3.66% and 2.87% Table 6 for 2002–2006 and 2004–2006, respectively.

**Table 6:** COPD confirmed cases and prevalence by periods and sex.

	2002–2006				2004–2006			
Prevalence	N	%^a^	95% CI		N	%^a^	95% CI	
**3-yrs-long estimates**	2098	3.66	3.65	3.67	1647	2.87	2.86	2.88
men	1384	6.19	6.16	6.22	1077	4.79	4.77	4.82
women	714	2.03	2.02	2.03	570	1.63	1.62	1.64
**5-yrs-long estimates**					1837	3.21	3.19	3.22
men					1205	5.37	5.34	5.39
women					632	1.81	1.8	1.82

COPD chronic obstructive pulmonary disease; 95% CI 95% confidence intervals.

^a^prevalence per 100 40+ year-old residents, standardized by age.

## Discussion

We found the highest estimate of COPD prevalence (4.43 per 100 residents) when we analyzed a 5-year period, used a 3-year longitudinal approach, and combined data from clinical charts and HD and CM registers. These choices allowed us to correct a 37% underestimation of COPD prevalence. We found 88% of confirmed cases among prevalent spirometry-tested COPDs, and estimated the validation coefficients for being an actual COPD case as 80% for the HDR, 82% for clinical diagnoses and 91% for deceased cases. These coefficients made it possible to correct 17% of misclassified COPD cases among all prevalent cases obtained from administrative data.

The global estimate of COPD prevalence was reported to be 10.1% (SE 4.8) in the BOLD study [1]. In Europe, estimates of COPD prevalence range from 10.2% in Spain to 26.1% in Austria, [19], while in Italy prevalence ranges from 4% to 6.7% in cities [20]. So—given these other estimates—underestimation may well still affect our results.

Among the factors influencing the variability of COPD prevalence, the most important were the criteria used to define COPD [6, 21] and the sources of data [22]. We defined COPD prevalence from administrative health databases by means of ICD-9 codes and the spirometric GOLD criteria based on a ratio of FEV1/FVC < 0.70. These criteria are the most sensitive of available classifications, including those of the British Thoracic Society, the European Thoracic Society and the American Thoracic Society [23, 13]. On the other hand, the fixed threshold makes it possible to compare estimates from many different countries and periods, given the high availability of these data worldwide [5, 13]. Our choice may involve some detriment to the specificity of COPD definition, since the fixed threshold of the FEV1/FVC ratio has been reported to overestimate airflow obstruction in 70+-year-olds [24]; in contrast, other studies [25] have shown that subjects in the in-between group (FEV1/FVC <0.7, but >LLN) had higher risks of hospitalization or mortality, suggesting a possible underestimation of airflow obstruction in the oldest cases when we used the LLN of FEV1/FVC. The sensitivity analysis we carried out here showed that the oldest confirmed cases of COPD decreased when we used LLN, but the interpretation of this is still in question.

Among the sources, hospitals contributed most cases (as both absolute and exclusive contributions) to prevalence estimates; however, outpatient data including spirometry registers testified to the great importance of non-hospitalized COPD cases in estimating prevalence, though the data from the other important local hospital were lacking. Nor could we include pharmaceutical data, which have been reported to contribute up to 55% of additional COPD cases (generally young and/or mildly affected) to those drawn from HDR and CMR [20]. The mortality register was the third source of cases with an important absolute contribution, i.e. 5% of cases. Constraints on access to the administrative databases are likely to have affected the sensitivity of our estimations as well as some patients’ recourse to private specialists. In addition, hospital and mortality databases are known to underestimate prevalence by about 20–60% [26], since they cover the most seriously ill patients. Mortality is specially affected because the concurrent causes of death may be reported instead of COPD [27]. Other countries use data from different health sources so as to include milder and well-managed COPD cases as well, as is the case with outpatient data in the United States of America [28] and general-practitioner data in the United Kingdom [8]. However, a few limitations affect these sources too: participation by general practitioners is usually voluntary and low [29], outpatient data do not always report diagnoses, and the difficulties inherent both in differentiating COPD treatment from asthma treatment and in fully validating pharmaceutical data [20].

Combining COPD data from different sources may increase the sensitivity of prevalence estimates for the following reasons. Underestimation of chronic diseases is intrinsic to most sources of health data, since performance in diagnosis and treatment differs among hospitals, emergency departments, outpatient clinics, physicians’ offices and prescriptions. In addition, the frequency of contact with chronic patients depends on the clinical course and treatment phase of a disease. Our results confirm these assumptions, showing that the longer the operation time of a database is and the greater the number of databases involved, the more cases of COPD may emerge, contributing to the estimates of the COPD burden. Other studies in the Netherlands [30] and Australia [26], show that combined data from different databases make it possible to estimate higher prevalence rates than can be drawn from a single source. Using multiple sources and multiple codes has also been shown to improve the accuracy with which COPD cases are identified from administrative databases [13, 18].

The recourse to spirometry was as low as 23% in our data, but not very different from that of other countries. Approximately 31–37% of COPD patients have spirometry in the USA [31–34] and in Canada [35]. The low proportion at our disposal was due to the absence of data from the other important local hospital; however, no selection bias seems to affect the patients who had spirometry compared with those who did not, among all those who were hospitalized, and using spirometric data from only one laboratory assured the high reproducibility of the tests. Estimates of up to 59% were reported recently in Sweden in a survey involving both primary and secondary care [36]. Spirometric testing was usually low among the oldest patients; it appears to decrease with increasing age in other studies as well, with the lowest frequency in > = 75-year-olds [32].

In contrast, the confirmation of COPD diagnosis reaches percentages as high as 88% in our study. Higher estimates (up to 92%) have been reported in Denmark [11], but among cases from the national COPD patient register. Values in administrative databases were more similar to our estimates: 89% is a recent estimate in the UK [12] when clinical diagnosis, spirometry and medication criteria were used to confirm COPD diagnoses. In Ontario (Canada), 85% was the confirmation estimate for outpatient and hospital administrative data according to an expert panel [37]. Finally, estimates of 87% (90–84% CI 95%) for HDR cases were reported in an Italian study [38].

The percentage of confirmed cases changes from source to among source. HDR merits more attention because its contribution is highest. Hospital cases identified by ICD-9 code 491.xx had the highest confirmation among the most specific codes for COPD (ICD-9 codes: 491.xx, 492.xx, 496.xx), as has been reported in the literature [7, 13, 37]. Two results are somewhat peculiar in our data: the lower COPD confirmation reported in the principal diagnosis than in a secondary one, and the highest confirmation for COPD as a secondary diagnosis with principal diagnoses which support a clinical worsening of COPD, such as heart failure or respiratory failure. The former result is confirmed in a recent paper from Ontario [39], which found 50.4% of confirmed principal diagnoses of COPD than secondary diagnoses. The latter result needs further study, as it suggests that reporting of COPD in hospital discharge registers could be improved.

The results of using only confirmed cases to estimate COPD prevalence in a population could be affected by a low recourse to spirometry. This is why using the coefficients of validation derived from the COPD cases observed at a specialized clinic, in this case the NRC-ICP, where 76% of patients had spirometry. Other experiences in as many periods and populations as possible are needed to confirm the method we propose here for correcting the misclassification of COPD prevalence estimates.

Our study is affected by a few important limitations. 1) The validation of COPD diagnoses relied on spirometric tests that lacked post-bronchodilator inhalation data, and this makes an overestimation of prevalence possible [6]. 2) The validation of COPD diagnoses also relied on reference values that were less reliable for 70+-year-olds. 3) The coefficients for validating the COPD cases reported in administrative databases were estimated in a small city.

## Conclusions

Combining data from different administrative databases may increase the sensitivity in estimating COPD prevalence, which is intrinsically lower with single sources. Applying validation coefficients of COPD diagnoses to the COPD prevalence estimates reduces the influence of misclassified cases. Increasing the use of post-BD spirometry in clinical practice, making spirometry results available for health administrative databases, and defining generally agreed-upon criteria for validating COPD cases are the next steps to be taken to make administrative databases reliable for epidemiological purposes.

## Supporting Information

S1 Table ICD-9 codes for COPD and co-morbidities. (DOCX)

## Abbreviations

BD: bronchodilator
CHF: congestive heart failure
95% CI: 95% confidence intervals
CMR: cause-specific mortality register
COPD: chronic obstructive pulmonary disease
FEV1: one-second forced expiratory volume
FVC: forced vital capacity
HDR: hospital discharge register
ICD-9: International Classification of Diseases, 9^th^ revision
ICP: Institute of Clinical Physiology
LLN: lower limit of normal
NRC: National Research Council
PPV: positive predictive value
RF: respiratory failure
SE: standard error
