# Correction: How Reliable Are Current Data for Assessing the Actual Prevalence of Chronic Obstructive Pulmonary Disease?

**DOI:** 10.1371/journal.pone.0159222

**Published:** 2016-07-08

**Authors:** Anna Maria Romanelli, Mauro Raciti, Maria Angela Protti, Renato Prediletto, Edo Fornai, Annunziata Faustini

There is an error in the “length of longitudinal periods” section of Table 1 in the third column. The correct value is “5yrs.” Please see the corrected Table 1 here.

**Table 1:** Algorithm for enrollment of COPD cases and contributing to COPD prevalence, according to prevalence periods and length of longitudinal periods.

	prevalence periods		
	2002–2006	2004–2006	2004–2006
	length of longitudinal periods		
	3yrs	3yrs	5yrs
Definition of cases	2000–2006	2002–2006	2000–2006
Subjects with one of the HDR ICD9 codes (490, 491, 492, 494, 496) as principal or secondary diagnosis and still alive at the beginning of the prevalence period	2182	1654	1897
+			
Subjects with COPD diagnosis in hospital chart, still alive at the beginning of the period, with no HDR report in the longitudinal period	17	12	13
+			
Subjects with COPD diagnosis in outpatient clinic chart, still alive and with no HDR report or hospital chart in the longitudinal period	33	35	33
+			
Subjects with spirometry and FEV1/FVC< = 0.70, with no HDR report or clinical charts in the longitudinal period	250	246	247
+			
Subjects deceased with COPD as underlined cause in the prevalence period, with no HDR report or clinical charts or spirometry in the longitudinal period	62	38	33
=			
**COPD prevalent cases enrolled in each period**	**2544**	**1985**	**2223**
