# Supplementary material for: How Reliable Are Current Data for Assessing the Actual Prevalence of Chronic Obstructive Pulmonary Disease?
Source: PLoS One. 2016 Feb 22;11(2):e0149302. doi: 10.1371/journal.pone.0149302 (PMC4763569; doi:10.1371/journal.pone.0149302)
Supplement: S1 Table — (DOCX) [file pone.0149302.s001.docx]

**S Table 1 Codes of International Classification of Diseases, 9^th^ revision (ICD-9)**

COPD

490.X bronchitis, not specified if acute or chronic

491.X chronic bronchitis

492.X emphysema

494.X bronchiectasis

496.X chronic obstruction of respiratory airways

not elsewhere classified

Respiratory failure

518.8 respiratory failure, acute or chronic

518.5 pulmonary insufficiency following trauma and surgery

786.0 dyspnea, sleep-obstructive apnea and other respiratory abnormalities

480-487 pneumonia

493 asthma

428.0 congestive heart failure
